# Improving *in vitro* induction efficiency of human primordial germ cell-like cells using N2B27 or NAC-based medium

**DOI:** 10.7555/JBR.38.20240433

**Published:** 2025-04-10

**Authors:** Gege Yuan, Jiachen Wang, Shuangshuang Qiu, Yunfei Zhu, Qing Cheng, Laihua Li, Jiahao Sha, Xiaoyu Yang, Yan Yuan

**Affiliations:** 1 State Key Laboratory of Reproductive Medicine and Offspring Health, Nanjing Medical University, Nanjing, Jiangsu 211166, China; 2 Women's Hospital of Nanjing Medical University, Women and Children's Healthcare Hospital, Nanjing, Jiangsu 211100, China; 3 State Key Laboratory of Reproductive Medicine, Clinical Center of Reproductive Medicine, the First Affiliated Hospital of Nanjing Medical University, Nanjing, Jiangsu 210029, China

**Keywords:** human primordial germ cell-like cell, N2B27, hypoxia, N-acetyl-L-cysteine

## Abstract

Primordial germ cells (PGCs), the precursors of oocytes and spermatozoa, are highly pluripotent. In recent years, the *in vitro* induction of human primordial germ cell-like cells (hPGCLCs) has advanced significantly. However, the stability and efficacy of obtaining hPGCLCs *in vitro* still require improvement. In the current study, we identified a novel induction system using Dulbecco's Modified Eagle Medium/Nutrient Mixture F-12 (DMEM/F-12) as the basal medium, supplemented with B27 and N2 (referred to as N2B27) in combination with four cytokines: bone morphogenetic protein 4, stem cell factor, epidermal growth factor, and leukemia inhibitory factor. The hPGCLCs induced under these conditions closely resembled PGCs from 4- to 5-week-old embryos at the transcriptomic level. Compared with traditional GK15 (GMEM supplemented with 15% Knockout™ Serum Replacement)-based induction conditions, the N2B27 system significantly increased the speed and efficacy of hPGCLC induction. RNA sequencing analysis revealed that this improvement was due to an increased cellular capacity to cope with hypoxic stress and avoid apoptosis. The N2B27 medium promoted enhanced mitochondrial activity, enabling cells to better manage hypoxic stress while reducing the production of reactive oxygen species. Moreover, through gradient concentration experiments, we demonstrated that the addition of the common antioxidant N-acetyl-L-cysteine at an optimized concentration further enhanced the efficiency of PGCLC induction under GK15 conditions. In summary, we have established an optimized induction system that enhances the efficiency of hPGCLC differentiation by improving cellular resilience to hypoxic stress and apoptosis.

## Introduction

Primordial germ cells (PGCs) are the precursor germ cells that emerge during early development and eventually give rise to mature sperm and oocytes^[1–3]^. PGCs first appear in the embryo around day 12 post-fertilization and subsequently migrate to the gonadal ridge, where they undergo further differentiation and proliferation, ultimately leading to the formation of mature germ cells^[4]^. Research on PGCs is crucial for improving our understanding of germ cell development and the mechanisms underlying infertility^[5–6]^.

Significant progress has been made in the study of mouse primordial germ cell-like cells, both in terms of system development and applications^[7–10]^. In recent years, the induction of human primordial germ cell-like cells (hPGCLCs) from pluripotent stem cells (PSCs) *in vitro* has also advanced considerably. Using factors, such as bone morphogenetic protein 4 (BMP4), stem cell factor (SCF), epidermal growth factor (EGF), and leukemia inhibitory factor (LIF), and methods, such as two-step induction and direct induction, investigators have obtained large quantities of seed cells for studies on germ cell development^[11–13]^. However, various methods exhibit instability, possibly due to the uncontrolled plasticity of PSCs in response to cytokines and the inherent limitations commonly encountered in *in vitro* experiments.

PSCs, particularly those sensitive to hypoxic stress, face significant challenges during the *in vitro* culture^[14]^. In contrast to the normoxic *in vitro* conditions (21% O_2_), the *in vivo* environment of PSCs is generally hypoxic, with oxygen levels ranging from 1% to 5%^[15–17]^. Culturing PSCs under normoxic conditions may induce oxidative stress, disrupting cellular metabolism and mitochondrial function^[18]^. Such an imbalance may lead to the accumulation of reactive oxygen species (ROS), which, if left unchecked, can cause oxidative damage. This increase in ROS impairs the proliferation, differentiation, and self-renewal of PSCs^[19]^. Therefore, it is essential to optimize the *in vitro* microenvironment to better mimic the physiological conditions of PSCs in order to reduce oxidative stress and preserve their functional integrity. Maintaining a balanced oxygen level and appropriate antioxidant presence in the culture system is crucial for ensuring the stability of PSC-related experiments.

In the current study, we developed a novel induction system for hPGCLCs using an N2B27 medium enriched with antioxidant components. We compared hPGCLC production under this new N2B27 system with the original GK15 induction conditions. Furthermore, the antioxidant N-acetyl-L-cysteine (NAC) was also tested within the GK15 framework to evaluate its effects.

## Materials and methods

### Cell source

The H1 human embryonic stem cells (H1-hESCs) used in the current study were purchased from the Chinese Academy of Sciences Stem Cell Culture Collection. All hESC studies were carried out following approval from the Institutional Review Board of Nanjing Medical University (Approval No. 2019935) and in accordance with the Ethical Guidelines for Human Embryonic Stem Cell Research issued by the Ministry of Science and Technology and the National Health Commission of the People's Republic of China, as well as the 2021 ISSCR Guidelines for Stem Cell Research and Clinical Translation.

### Culture of human PSCs (hPSCs)

The hPSCs were cultured under the following conditions: 37 ℃, 5% CO_2_, and saturated humidity. The H1-hESCs were maintained in a feeder-free system using Essential 8^TM^ medium (Cat. #A1517001, Gibco, Waltham, MA, USA). Cells were plated in six-well plates (Cat. #353046, Corning, NY, USA) pre-coated with Matrigel Matrix (Cat. #354230, Corning) according to the manufacturer's protocol. H1-hESCs were used before passage 55, while DYR0100-hiPSCs were used before passage 40.

### hPGCLC induction

To induce hPGCLC aggregates, the following conditions were used: 37 ℃, 5% CO_2,_ and saturated humidity. As reported previously^[12]^, hPSCs were first induced into human incipient mesoderm-like cells (iMeLCs) and then into hPGCLCs. The hPSCs were treated with TrypLE Select (Cat. #12563-011, Thermo Fisher Scientific, Waltham, MA, USA) to dissociate them into single cells. Subsequently, the cells were seeded at a density of (7.5–8.5) × 10^5^ cells per well onto the fibronectin-coated (Cat. #FC010, Millipore, Billerica, MA, USA) six-well plate in either GK15 or N2B27 media supplemented with 50 ng/mL activin A (Cat. #338-AC-500, R&D Systems, Minneapolis, MN, USA), 100 ng/mL WNT3A (Cat. #5036-WN-500, R&D Systems), and 10 μmol/L of Y-27632 (Cat. #HY-10583, MedChemExpress, Monmouth Junction, NJ, USA). The GK15 medium contained Glasgow's Minimum Essential Medium (GMEM; Cat. #11710035, Thermo Fisher Scientific) with 15% KnockOut™ Serum Replacement (KSR; Cat. #10828028, Thermo Fisher Scientific), 0.1 mmol/L non-essential amino acid (NEAA; Cat. #11140-050, Thermo Fisher Scientific), and 0.1 mmol/L 2-mercaptoethanol (Cat. #21985-023, Thermo Fisher Scientific). The N2B27 medium contained Dulbecco's Modified Eagle Medium/Nutrient Mixture F-12 (DMEM-F12; Cat. #11330032, Thermo Fisher Scientific) supplemented with N2 (Cat. #17502001, Thermo Fisher Scientific), B27 (Cat. #17504044, Thermo Fisher Scientific), 0.1 mmol/L NEAA, and 0.1 mmol/L 2-mercaptoethanol.

hPSCs were treated with TrypLE Express (Cat. #12605028, Gibco) at 37 ℃ for 3 min, followed by gentle mechanical dissociation using a pipette. After centrifugation, the cell pellets were resuspended in GK15 or N2B27 media, supplemented with 200 ng/mL hBMP4 (Cat. #314-BP, R&D Systems), 100 ng/mL hSCF (Cat. #BT-SCF, R&D Systems), 50 ng/mL hEGF (Cat. #236-EG, R&D Systems), 1000 U/mL hLIF (Cat. #7734-LF, R&D Systems), and 10 μmol/L of Y-27632 to induce hPGCLCs. Next, 1.5 × 10^6^ cells were mixed with 2 mL of culture medium and seeded into one well of the 12-well AggreWell^TM^ 800 culture plate (Cat. #43815, Stem Cell Technologies, Vancouver, BC, Canada) pretreated with an anti-adherence rinsing solution (Cat. #07010, Stem Cell Technologies) following the manufacturer's instructions. The cells were allowed to form aggregates within the microwell for 24 h. Then, we gently aspirated the aggregates and transferred them to low-adhesion, six-well plates (Cat. #3471, Corning) with 4 mL of medium devoid of Y-27632 per well for continued culture. The medium was refreshed every 24 h, with 75% of the medium changed each time.

### Fluorescence-activated cell sorting (FACS) and flow cytometry (FCM)

Once washed in phosphate-buffered saline (PBS), the hPGCLC aggregates were dissociated with TrypLE at 37 ℃ for 15 min. Then, epithelial cell adhesion molecule (EpCAM; 1∶50, Cat. #369809, BioLegend, San Diego, CA, USA) and integrin subunit alpha 6 (ITGA6; 1∶50, Cat. #313612, BioLegend) antibodies were incubated with the dissociated cells at 37 ℃ for 20 min. Cells expressing high levels of EpCAM and ITGA6 were sorted using a FACSAria Fusion system (San Jose, CA, USA). Three independent experimental replicates under each condition were included in the statistical analysis.

### Immunofluorescence staining

The samples were fixed with 4% paraformaldehyde in PBS at room temperature for 20 min, washed three times with Dulbecco's Phosphate-Buffered Saline (DPBS; Cat. #R21-031-CV, Corning), and permeabilized with 1% Triton X-100 (Cat. #A110694, Sangon, Shanghai, China). After blocking with 5% bovine serum albumin (Cat. #V900933, Sigma-Aldrich, St. Louis, MO, USA) in DPBS (with 1% Triton X-100) at room temperature for 4 h, samples were incubated with a primary antibody diluted in blocking buffer at 4 ℃ for 24 h. After incubation with the primary antibodies, samples were washed three times with DPBS and incubated with fluorescence-conjugated secondary antibodies diluted in blocking buffer at room temperature for 4 h. Nuclei were stained with Hoechst (1∶200, Cat. #62249, Gibco). Samples were washed three times with DPBS and prepared for imaging.

The primary antibodies included goat anti-SOX17 (1∶500, Cat. #AF1924, R&D Systems), mouse anti-AP-2 gamma (1∶100, Cat. #SC-12762, Santa Cruz Biotechnology, Dallas, TX, USA), and rabbit anti-BLIMP1 (1∶200, Cat. #9115, Cell Signaling Technology, Danvers, MA, USA). The secondary antibodies (Invitrogen, Waltham, MA, USA) included Alexa Fluor 488 donkey anti-mouse (1∶200, Cat. #A-21202), Alexa Fluor 488 donkey anti-rabbit (1∶200, Cat. #A-21206), Alexa Fluor 555 donkey anti-mouse (1∶200, Cat. #A-31570), Alexa Fluor 555 donkey anti-rabbit (1∶200, Cat. #A-31572), and Alexa Fluor 647 donkey anti-goat (1∶200, Cat. #A-21447).

### Sample clearing

Twenty grams of Histondenz^TM^ (Cat. #D2158, Sigma-Aldrich) was dissolved in 15 mL of 0.2× PBS for long-term storage at room temperature.

For sample clearing, 45 μL of the clearing solution was added to the CoverWell^TM^ incubation chamber gaskets (Cat. #C18155, Thermo Fisher Scientific), then cleaned samples (after incubation with the secondary antibody) were transferred to chambers. After gently mixing the samples well in the clearing solution using a mouth pipette, we slowly covered the surface of the coverslip from the incubation chamber gasket side, minimizing bubble formation. The fixed sample was placed in the dark until it was completely cleared.

### Microscopy and image analysis

Brightfield images were taken with an inverted fluorescence microscope TE2000-s (Nikon, Shinagawa, Tokyo, Japan) equipped with an AxioCam HRc camera (Zeiss, Oberkochen, Germany) and another inverted microscope, Eclipse Ti2-U (Nikon). Confocal immunofluorescence images of hPGCLC aggregates were acquired using a Zeiss LSM 800 confocal microscope. Images were processed using ZEN (Zeiss).

### Cell growth status of aggregates

Cells were seeded at a density of 1.5 × 10^6^ cells per well under hPGCLC induction conditions (including both GK15 and N2B27 induction conditions) in six replicates. Aggregates were collected at 24-h intervals for FCM analysis. At each time point, cells were digested and counted using the Countess Ⅱ Automated Cell Counter (Thermo Fisher Scientific).

### Mitochondrial health and membrane potential analysis

Once washed in PBS, the hPGCLC aggregates were dissociated with TrypLE at 37 ℃ for 15 min. Then, 20 nmol/L MitoTracker Green (MTG, Cat. #M7514, Thermo Fisher Scientific) and 20 nmol/L Tetramethylrhodamine Methyl Ester (TMRM, Cat. #I34361, Thermo Fisher Scientific) were incubated with the dissociated cells at 37 ℃ for 30 min. After incubation, the cells were washed three times with DPBS to remove excess dye. Cells stained with MTG and TMRM were analyzed using the FACSAria Fusion system. Three independent replicates under each condition were included in the statistical analysis.

### ROS assay

A ROS Assay Kit (Cat. #50101ES01, Yeasen, Shanghai, China) was used for this experiment. Once washed in PBS, the hPGCLC aggregates were dissociated with TrypLE at 37 ℃ for 15 min. The appropriately diluted probe (10 μmol/L) was added and incubated with the cells in a 37 ℃ incubator for 30 min. The samples were mixed gently every 3–5 min to ensure thorough contact between the probe and the cells. After incubation, the cells were washed three times with DPBS to remove excess dye. The incubated cells were analyzed using the FACSAria Fusion system.

### NAC gradient experiment

The antioxidant NAC (Cat. #HY-B0215, MedChemExpress) was set in groups of 0, 0.25, 0.50, 0.75, and 1.00 mmol/L. NAC was added continuously during hPGCLC induction under GK15 conditions, and corresponding analyses were performed four days after sampling.

### RNA sequencing (RNA-Seq) sample collection and library preparation

Total RNA was extracted from human PGCLC induced from H1-hESCs using the FreeZol Reagent (Cat. # R711-02, Vazyme, Nanjing, China), following the manufacturer's protocol. For transcriptome sequencing, 1 μg of total RNA per sample was used as input material. Sequencing libraries were prepared using the NEBNext® Ultra™ RNA Library Prep Kit for Illumina® (NEB, USA) according to the manufacturer's protocol. Index codes were incorporated into each sample to facilitate sequence identification. The clustering of index-coded libraries was performed on a cBot Cluster Generation System using the TruSeq PE Cluster Kit v3-cBot-HS (Illumina, USA) according to the manufacturer's instructions. Following cluster generation, the libraries were sequenced on an Illumina NovaSeq platform, generating 150 bp paired-end reads. Sequencing data quality was assessed using FastQC (v0.11.5) to ensure adequate read quality, and adapter contamination levels were monitored.

### Bioinformatic analysis of RNA-Seq

The paired-end clean reads were aligned to the human reference genome (GRCh38, GENCODE annotation release v34) using HISAT2 (Version 2.1.0)^[20]^ with default parameters. The gene-level read counts were quantified using featureCounts (Version 2.0.0)^[21]^ with the GTF annotation file from GENCODE v34. Only uniquely mapped reads were included in the downstream analysis to ensure the accuracy of gene expression estimates.

Transcript abundance was calculated as transcripts per million (TPM), and all subsequent analyses were based on log_2_(TPM + 1)-transformed values. Differential expression analysis was performed using the DESeq2 package (Version 1.36.0)^[22]^ within the R software environment (Version 3.6.3). Genes were considered differentially expressed if they met the thresholds of |log_2_(fold change)| > 1 and an adjusted *P*-value < 0.05, as calculated using the Benjamini-Hochberg method to control for false discovery rates.

To visualize data variability and sample clustering, principal component analysis (PCA) was performed using the prcomp function in R. Heatmaps of differentially expressed genes (DEGs) were generated using the ComplexHeatmap package (Version 2.15.3)^[23]^, and other visualizations were constructed using ggpubr (Version 0.4.0) and tidyverse (Version 1.3.0). For additional 3D visualizations of expression patterns, the rgl (Version 0.100.54) package was employed.

Functional enrichment analyses, including Gene Ontology (GO) and Kyoto Encyclopedia of Genes and Genomes (KEGG) pathway analyses, were conducted using the DAVID bioinformatics web tool^[24]^. Results of GO and KEGG enrichment analyses were filtered using a significance threshold of *P*-value < 0.05.

The public datasets used in the current study are available from the Gene Expression Omnibus (GEO) database under accession numbers GSE60138, GSE67259, and GSE86146. The data generated in the current study will be made publicly available in a suitable repository upon the manuscript's acceptance.

### Statistical analysis

Student's *t*-tests were used for comparisons between two groups. These analyses were performed using GraphPad Prism Version 8.0 software (GraphPad Software, San Diego, CA, USA). All data points collected in the current study were included in the statistical analyses. Data were presented as the mean ± standard error of the mean, and a *P*-value of less than 0.05 was considered statistically significant.

## Results

### Induction of aggregates with PGCLC characteristics *via* the N2B27 medium

In recent years, hPSCs have been used to induce hPGCLCs *in vitro* *via* a two-step approach with different basal media (aRB27 and GK15) containing different cytokines^[11–12]^. Building on these published methods, we aimed to optimize and develop more effective hPGCLC induction protocols *in vitro*. We performed tests with four combinations of basal media, including the traditional GK15 medium (GMEM and 15% KSR) for inducing hPGCLCs and the optimized N2B27 medium (DMEM/F12 supplemented with N2 and B27), which had not been previously used for hPGCLC induction (***Supplementary Fig. 1A***, available online).

**Figure 1:**
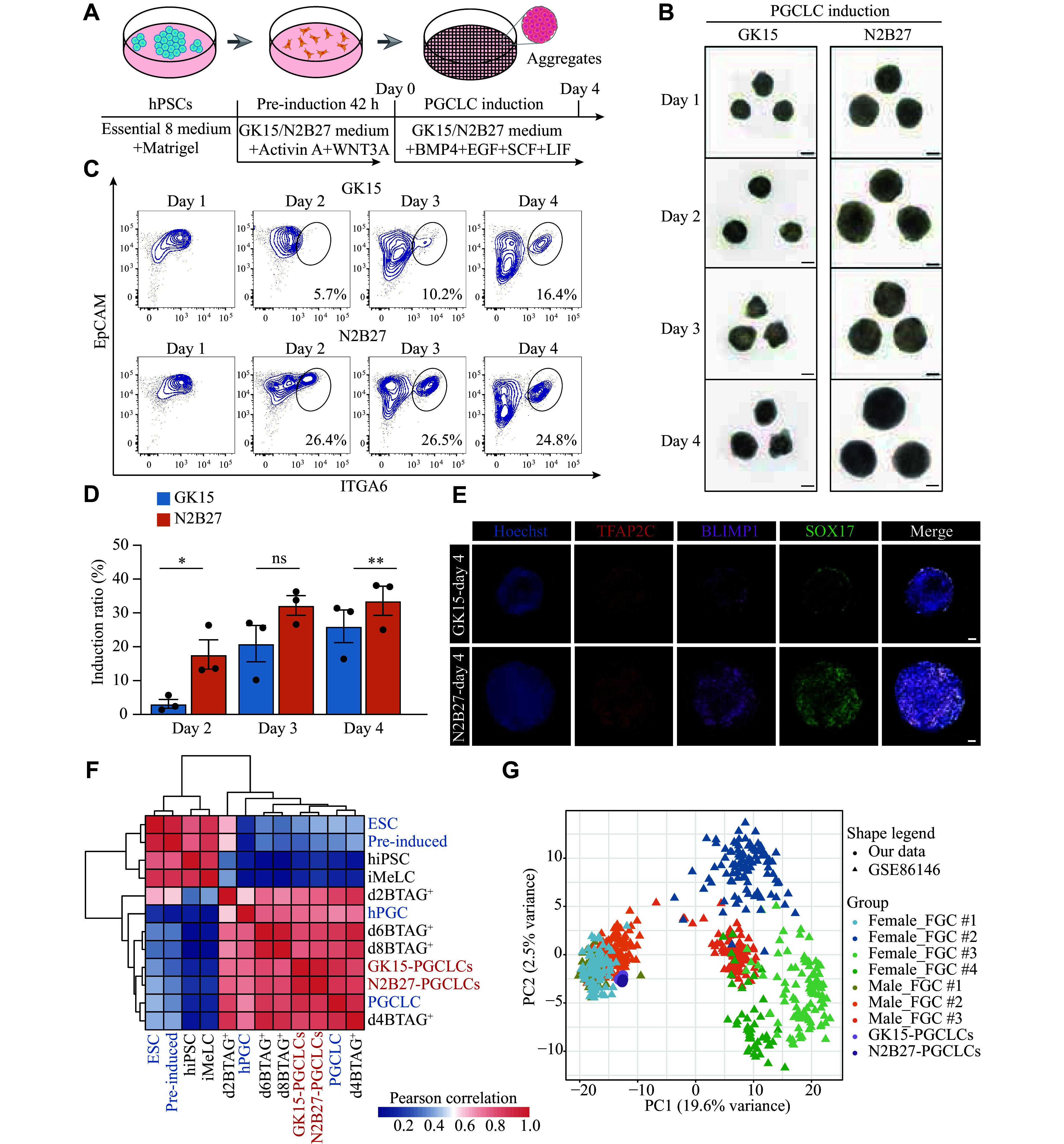
Induction of aggregates with PGCLC characteristics *via* the N2B27 medium. A: Schematic of the hPGCLC induction from hPSCs. B: Representative bright-field images of floating aggregates during hPGCLC induction from day 1 to day 4 under GK15 (left) or N2B27 (right) conditions. Scale bars, 100 μm. C: Flow cytometry analysis of EpCAM and ITGA6 expression during the hPGCLC induction from day 1 to day 4 under GK15 (up) or N2B27 (bottom) induction conditions. D: Histogram presenting the variation in the proportion of EpCAM and ITGA6 double-positive cells under GK15 (blue) and N2B27 (orange) induction conditions from day 2 to day 4. Data are presented as mean ± standard error of the mean. ^*^P < 0.05, ^**^P < 0.01, and ^ns^*P* > 0.05 by unpaired two-sided Student's *t*-test. The results from three experiments under each condition are shown. E: Immunofluorescence staining for the primordial germ cell marker SOX17 (green), TFAP2C (red), and BLIMP1 (pink) in aggregates on day 4 under GK15 (up) or N2B27 (bottom) induction conditions. Nuclei were counterstained with Hoechst (cerulean blue). Scale bars, 50 μm. F: Heatmap of correlation coefficients among cells, including EpCAM and ITGA6 double-positive cells under GK15 and N2B27 induction conditions on day 4 (red), alongside cell types from GSE60138 (blue) and GSE67259 (black). Types from GSE67259 are hiPSC, iMeLC, and the BLIMP1-2A-tdTomato and the TFAP2C-2A-EGFP cells (BTAG^+^) induced through iMeLCs (days 2, 4, 6, and 8). Types from GSE60138 are 4i hESC, preinduced cells (pre-induced), day 4 hPGCLCs (PGCLC), and gonadal hPGC (hPGC). G: Principal component analysis of the indicated cell types, including EpCAM and ITGA6 double-positive cells under GK15 and N2B27 induction conditions on day 4, alongside cell types from GSE86146. The cells (color-coded as indicated) are plotted in a two-dimensional space defined by PC1 and PC2. Abbreviations: hPSCs, human pluripotent stem cells; hPGCLCs, human primordial germ cell-like cells; FGC, fetal germ cell; hiPSC, human induced pluripotent stem cells; iMeLC, incipient mesoderm-like cells; hPGC, human primordial germ cell; BMP4, bone morphogenetic protein 4; EGF, epidermal growth factor; SCF, stem cell factor; LIF, leukemia inhibitory factor; EpCAM, epithelial cell adhesion molecule; ITGA6, integrin alpha-6; TFAP2C, transcription factor AP-2 gamma; SOX17, SRY-Box transcription factor 17; BLIMP1, B lymphocyte-induced maturation protein 1.

First, hPSCs were pre-induced into iMeLCs for 42 h in the N2B27 medium supplemented with WNT3A and activin A. Then, iMeLCs were further induced into PGCLCs in the N2B27 medium containing BMP4, SCF, LIF, EGF, and a ROCK inhibitor under floating aggregate conditions (***Fig. 1A***). Bright-field imaging revealed that the aggregates exhibited the largest size under combination 1, followed by combination 2 (***Supplementary Fig. 1B***, available online). Using FCM analysis with the known surface markers (EpCAM and ITGA6) of PGCLCs on day 4 of the aggregate induction, we found that the PGCLC induction efficiency was greater under combination 1, followed by combination 2 (***Supplementary Fig. 1B***). Therefore, we performed further tests using the N2B27 medium for inducing both iMeLCs and hPGCLCs, with GK15 medium used as the control.

Bright-field imaging on day 1 showed that aggregates were larger in the N2B27 medium than in the GK15 medium, indicating that iMeLCs cultured under these conditions had a greater anti-apoptotic capacity. Aggregates in N2B27 showed more noticeable proliferation throughout the induction process (***Fig. 1B***). The number of proliferating aggregates in GK15 and N2B27 media during induction also supported this observation (***Supplementary Fig. 1C***, available online). Through FCM analysis of aggregates from days 1 to 4, we found that the PGCLC induction efficiency was greater under N2B27 than under GK15 culture conditions (***Fig. 1C*** and ***1D***). A distinct EpCAM^+^ and ITGA6^+^ population appeared as early as day 2 under N2B27 culture conditions, whereas population clustering was not observed until day 3 under GK15 culture conditions (***Fig. 1C***).

To verify that N2B27 culture conditions are more conducive to PGCLC induction, we performed immunofluorescence staining on the day 4 aggregates using known PGC markers (SOX17, BLIMP1, and TFAP2C). Under N2B27 culture conditions, cells co-expressing SOX17, BLIMP1, and TFAP2C were scattered throughout the aggregates. In contrast, under GK15 culture conditions, these triple-positive cells were more concentrated in the outer layer rather than in the center of the aggregates (***Fig. 1E***).

To further characterize the induced hPGCLCs, we sorted EpCAM^+^ and ITGA6^+^ cells using FACS, performed bulk RNA-seq, and compared these results with previously published data^[11–12]^. We performed Pearson correlation analysis of the DEGs among all the cell types. The clustering analysis further supported that the EpCAM/ITGA6 double-positive cells had the highest correlation with day 4 PGCLC from the published data (***Fig. 1F***). PCA results further corroborated this consistency (***Supplementary Fig. 1D***, available online). Among the relevant cell types from the two-step induction process, the EpCAM/ITGA6 double-positive cells presented the fewest DEGs [log_2_(TPM + 1) > 4 and log_2_(fold change) > 2], compared with the BLIMP1-2A-tdTomato and TFAP2C-2A-EGFP double-positive cells on day 4 (d4BTAG^+^) in both culture media (***Supplementary Fig. 1E*** and ***Supplementary Table 1***, available online). The expression levels of early PGC markers (*SOX17*, *TFAP2C*, and *BLIMP1*) and germ layer-associated genes (*EOMES*, *GATA4*, and *GATA6*) were comparable between the EpCAM/ITGA6 double-positive cells and the d4BTAG^+^ cells. However, in the EpCAM/ITGA6 double-positive cells, the expression levels of pluripotency-related genes (*NANOS3*, *NANOG*, and *POU5F1*) were expressed at higher levels (***Supplementary Fig. 1F***, available online). These findings suggest that the hPGCLCs derived under these conditions exhibited robust pluripotency, partially resembling a naïve pluripotency state. These observations are consistent with previous reports^[25–26]^.

To further evaluate the agreement between the *in vitro*-induced hPGCLCs and the *in vivo* developmental processes, we compared hPGCLCs derived from both culture media (GK15 and N2B27) with single-cell transcriptomic profiles of fetal germ cells (FGCs) throughout embryonic development^[27]^. The PCA results revealed a directional and progressive transition in cellular properties during FGC development. Notably, hPGCLCs presented a greater similarity to the cell cluster containing FGC-1 (female) and FGC-1/2 (male) cells (***Fig. 1G***). In contrast, the differences between hPGCLCs derived from N2B27 and those derived from GK15 were negligible. To determine which embryonic stage of FGCs-1/2 was more similar to that of hPGCLCs derived from the GK15 and N2B27 conditions, we conducted a similarity analysis. The unsupervised hierarchical clustering revealed that our hPGCLCs were most similar to male FGC-1 cells from four-week-old embryos (***Supplementary Fig. 1G***, available online). PCA showed similar results (***Supplementary Fig. 1H***, available online).

These findings suggest that the cultivated cells with PGC characteristics and the EpCAM/ITGA6 double-positive cells during induction could be defined as hPGCLCs, with a relatively high induction efficiency achieved under N2B27 culture conditions.

### N2B27 accelerated the hPGCLC induction rate

**Figure 2:**
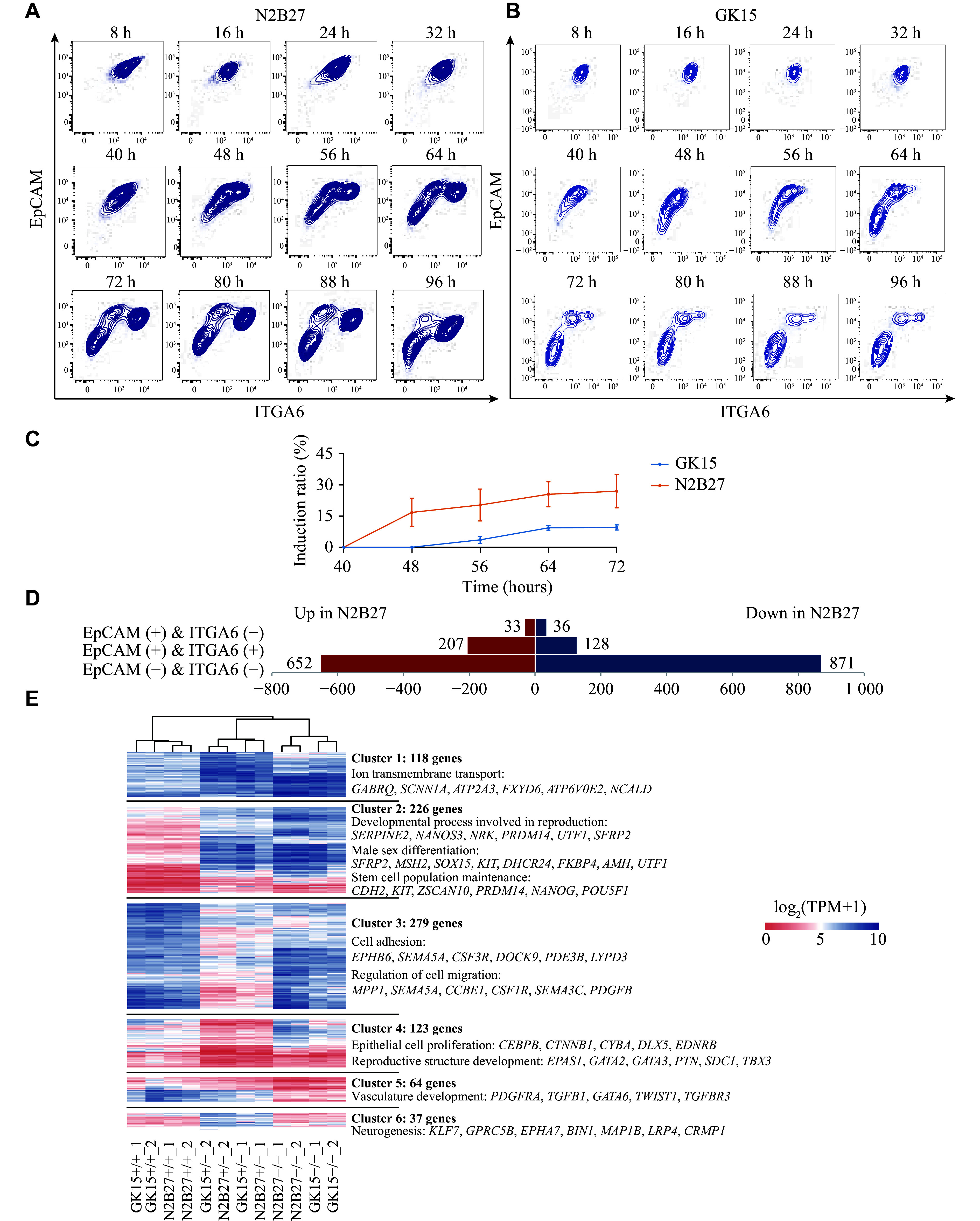
N2B27 medium accelerated the hPGCLC induction rate. A and B: Flow cytometry analysis of EpCAM and ITGA6 expression during hPGCLC induction from day 0 to day 4 under N2B27 (A) and GK15 (B) conditions, with measurements taken every 8 h. C: Line graph presenting the variation in the proportion of EpCAM and ITGA6 double-positive cells under GK15 (blue) and N2B27 (orange) induction conditions from 40 h to 72 h. D: Number of differentially expressed genes in EpCAM-positive/ITGA6-weakly positive (+/−), EpCAM/ITGA6 double-positive (+/+), and EpCAM/ITGA6 double-negative (−/−) cells derived from GK15 and N2B27 induction conditions. E: Heatmap showing the differentially expressed genes among EpCAM-positive/ITGA6-weakly positive (+/−), EpCAM/ITGA6 double-negative (−/−), and EpCAM/ITGA6 double-positive (+/+) cells derived from GK15 and N2B27 induction conditions. Abbreviations: EpCAM, epithelial cell adhesion molecule; ITGA6, integrin alpha-6; hPGCLCs, human primordial germ cell-like cells.

We next examined the dynamics of hPGCLCs derived from two induction conditions. FCM was performed every 8 h during the hPGCLC induction under GK15 and N2B27 conditions *via* the PGC surface markers ITGA6 and EpCAM. EpCAM and ITGA6 have previously been reported to be expressed on hPSCs, PGCLCs, and other cell types^[28]^. Under N2B27 induction conditions, the EpCAM/ITGA6 double-positive cell population, representing hPGCLCs, emerged between 40 h and 48 h, at which point three distinct cell types were identified (***Fig. 2A*** and ***2C***). Under GK15 induction conditions, the appearance of the same cell population was delayed, occurring at about 48–64 h. During the subsequent period, the proportion of EpCAM/ITGA6 double-positive (+/+) cells progressively increased, with a more prominent separation from other cell populations (***Fig. 2B*** and ***2C***). The differentiation process was described as the transition of the EpCAM-positive/ITGA6-weakly positive (+/−) cells to the EpCAM/ITGA6 double-negative (−/−) and the EpCAM/ITGA6 double-positive (+/+) cells.

We detected transcriptomic differences among the three cell populations under N2B27 and GK15 conditions. The differences between EpCAM-positive/ITGA6-weakly positive (+/−) cells in the two media were minimal, whereas significant differences were found among the double-negative (−/−) cells (***Fig. 2D***). These results suggest that within aggregates, there is considerable variability in the differentiation of non-PGCLC cell types, which may influence PGCLC generation.

We separately analyzed the differences between the three cell types derived from the GK15 and N2B27 conditions and merged them to obtain 847 DEGs (***Fig. 2E***). The findings indicated that these genes were significantly different in the three cell types regardless of the medium. The unsupervised hierarchical clustering classified these genes into six clusters, with each cluster showing specific expression in distinct cell types. The genes in clusters 1 and 2 (118 and 226 genes, respectively), especially those in cluster 2 with relatively high expression levels, were specifically expressed in the EpCAM/ITGA6 double-positive cells and enriched for GO terms such as "stem cell population maintenance", "male sex differentiation", and "developmental process involved in reproduction". In contrast, the genes in cluster 5 (64 genes) exhibited specific enriched expression in the EpCAM/ITGA6 double-negative cells and were enriched with GO terms related to the blood and vasculature, such as "vasculature development". Consistent with previous results, the genes in clusters 3 and 4 (279 and 123 genes, respectively) were enriched with GO terms correlated with epithelial characteristics and cell adhesion and migration. Genes in cluster 6 (37 genes) presented common characteristics of double-negative cells and were enriched with GO terms such as "neurogenesis" (***Fig. 2E***; ***Supplementary Table 2*** [available online]). These findings suggest that within aggregates, the EpCAM-positive/ITGA6-weakly positive (+/−) and the EpCAM/ITGA6 double-negative (−/−) cell populations may be correlated with the development of the hematopoietic system, neural cells, or epithelial cells, resembling the multilineage cell fate observed in undirected induced embryoid bodies^[29]^. Stem cells exhibit plasticity and uncertainty in response to various cytokines. Under N2B27 induction conditions, hPGCLCs were differentiated more readily within the aggregates. In contrast, under GK15 induction conditions, even with the same cytokines and concentrations, differentiation was biased toward non-hPGCLC cell types.

These results demonstrated that under N2B27 induction conditions, hPGCLCs were generated earlier, ensuring that hPSCs differentiated into hPGCLCs rather than other cell types that also responded to LIF, EGF, SCF, and BMP4.

### Generation of difference between hPGCLCs from two media focused on hypoxia

**Figure 3:**
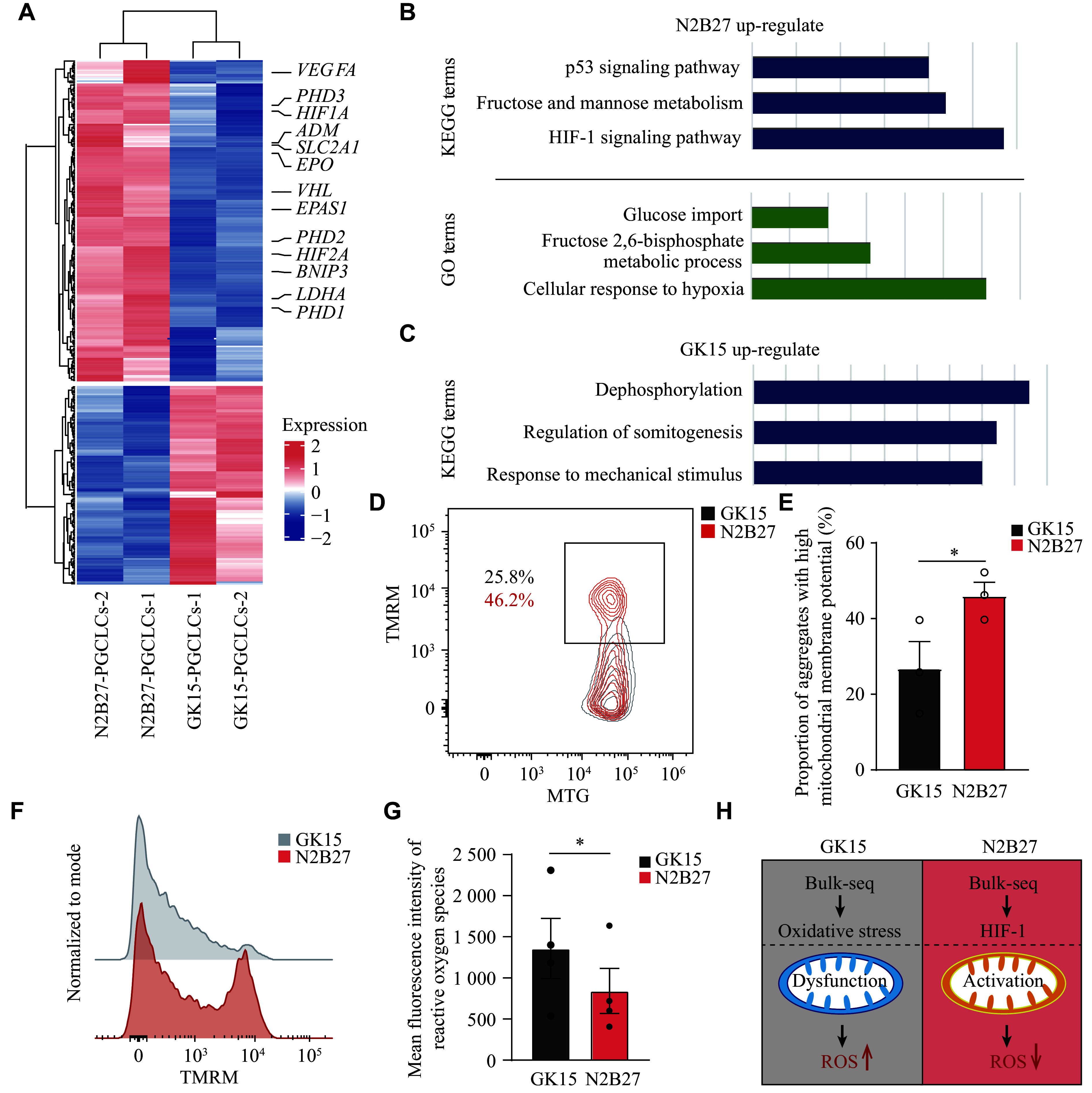
Generation of difference between hPGCLCs from two media focused on hypoxia. A: Heatmap showing the differentially expressed genes of hPGCLCs between GK15 and N2B27 induction conditions. B: KEGG and GO analysis of the up-regulated genes in hPGCLCs under N2B27 induction conditions in ***Fig. 3A***. C: KEGG analysis of the up-regulated genes in hPGCLCs under GK15 induction conditions in ***Fig. 3A***. D: Flow cytometry analysis of mitotracker green (MTG) and tetramethyl rhodamine methylester (TMRM) expression in aggregates on day 4 under GK15 (grey) or N2B27 (red) conditions. E: Histogram presenting the proportion of cells in aggregates with high mitochondrial membrane potential on day 4 under GK15 (grey) or N2B27 (red) conditions. F: Representative flow plot showing the distribution of TMRM fluorescence intensity of aggregates under GK15 (grey) or N2B27 (red) induction conditions. G: Histogram presenting the average fluorescence intensity of ROS expression in aggregates on day 4 under GK15 (grey) or N2B27 (red) induction conditions. H: Schematic of cells in GK15 (left) and N2B27 (right) induction conditions: mitochondrial status and ROS status. Data are presented as mean ± standard error of the mean. ^*^P < 0.05 by unpaired two-sided Student's *t*-test. *n* = 3 for E, and *n* = 4 for G. Abbreviations: hPGCLCs, human primordial germ cell-like cells; ROS, reactive oxygen species.

We next investigated the underlying reasons for the differences in hPGCLC induction under two different culture conditions. To identify the global gene expression characteristics of hPGCLCs derived from the two media, we compared their transcriptome data. Among the genes whose expression was upregulated in hPGCLCs cultured in the N2B27 medium, many, such as *PDH*, *HIF1A*, *SLC2A1*, *PHD2*, and *DNIP3*, are involved primarily in the cellular hypoxic response system, regulating the hypoxia-inducible factor (HIF) pathway (***Fig. 3A***; ***Supplementary Table 3*** [available online]), which helps cells adapt to hypoxic or oxidative stress environments^[30–32]^. Both GO and KEGG analyses were subsequently performed on the DEGs. The results revealed that significant biological pathways, such as fructose and mannose metabolism, the HIF-1 signaling pathway, and the response to oxygen levels, were enriched under N2B27 culture conditions (***Fig. 3B*** and ***3C***; ***Supplementary Table 3***). These results suggest that under N2B27 induction conditions, PGCLCs responded more actively to the *in vitro* environment, thereby mitigating potential negative effects on development. This may be because the N2B27 culture environment is rich in antioxidants from the DMEM-F12 medium and B27 supplements, whereas the GK15 culture environment lacks these components to a similar extent. Under the GK15 condition, PSCs appear inadequately equipped to manage the hypoxic environment, resulting in oxidative stress and mitochondrial dysfunction.

Previous studies have indicated that during PGCLC induction from PSCs, energy production depends not only on glycolysis but also requires oxidative phosphorylation, necessitating mitochondrial activation^[33–35]^. To investigate mitochondrial activity differences in aggregates under the two culture conditions, MTG was used to indicate the total mitochondrial pool, while TMRM labeled active mitochondria with intact membranes. The MTG and TMRM-labeled cells were analyzed through FCM. A higher proportion of TMRM-positive cells was observed under the N2B27 condition than the GK15 condition (***Fig. 3D*** and ***3E***). Furthermore, TMRM-positive cells from N2B27 induction conditions exhibited a generally higher mitochondrial membrane potential, indicating that N2B27 conditions provide a better protection for mitochondrial homeostasis (***Fig. 3F***). This observation suggests that the induction process under N2B27 conditions successfully transitions to oxidative phosphorylation for energy production, whereas energy production under the GK15 condition remains relatively insufficient, adversely affecting the PGCLC generation efficiency.

Additionally, ROS levels were examined, as their accumulation occurs not only under hypoxia but also as a result of mitochondrial dysfunction^[36–37]^. ROS levels were compared between aggregates derived from different induction conditions, revealing significantly lower ROS levels under N2B27 than under GK15 induction conditions (***Fig. 3G***).

These results indicate that the aggregates under GK15 and N2B27 induction conditions respond differently to hypoxia during induction, which may be due to the presence of a greater number of antioxidants under N2B27 culture conditions (***Fig. 3H***).

### Effect of NAC on enhancing the induction efficiency of PGCLCs

The experimental results suggested that antioxidants might enhance the *in vitro* induction of PGCLCs. To further investigate this phenomenon, we added antioxidants under GK15 induction conditions to evaluate their ability to improve the PGCLC induction efficiency. We identified NAC, an antioxidant that targets the HIF-1 pathway, which can reduce oxidative stress and enhance mitochondrial bioenergetics^[38–39]^.

**Figure 4:**
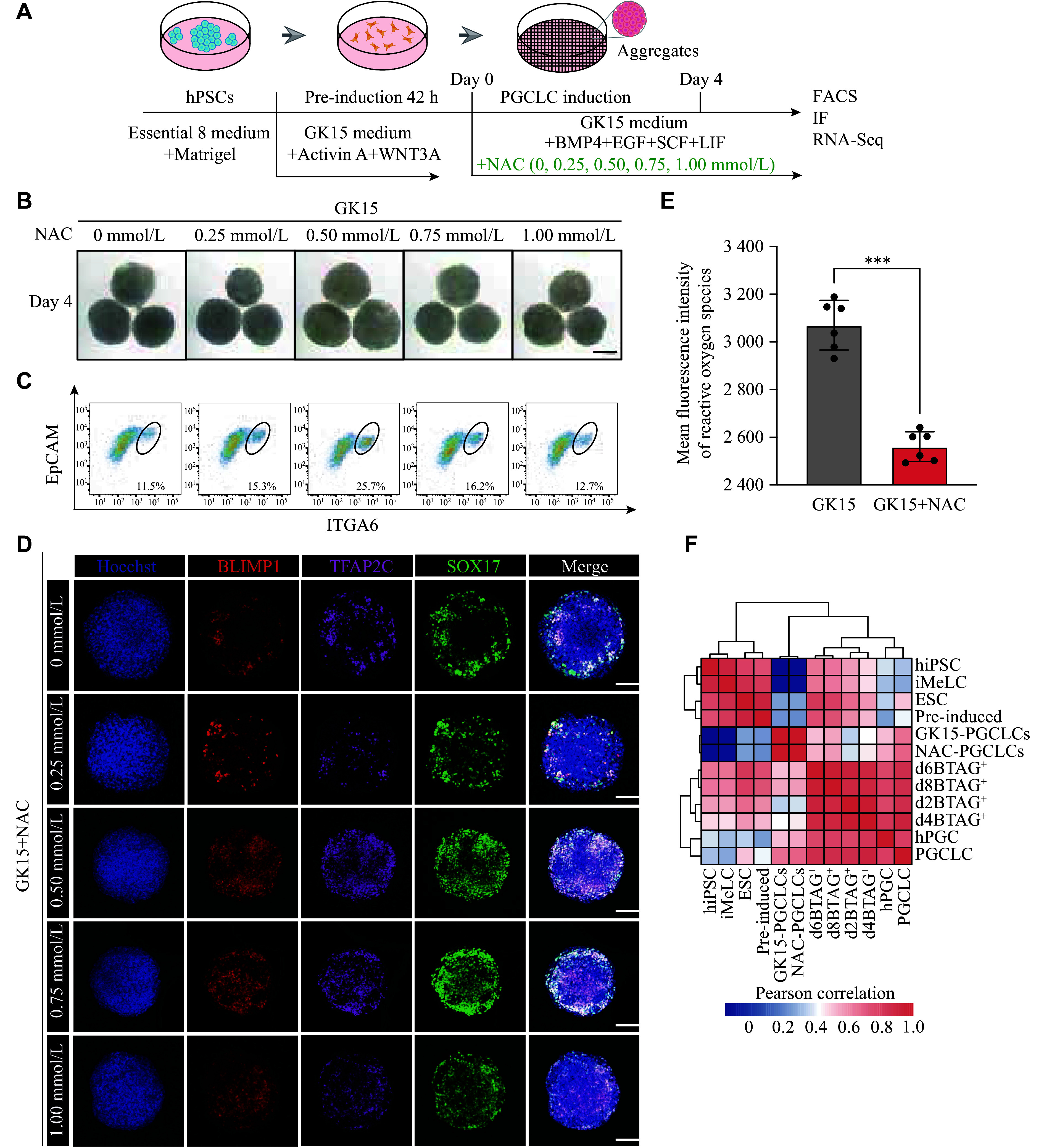
Effect of NAC on enhancing the induction efficiency of PGCLCs. A: Schematic of hPGCLC induction under GK15 conditions supplemented with NAC from hPSCs. B: Representative bright-field images of floating aggregates on day 4 during hPGCLC induction under GK15 conditions supplemented with NAC (0, 0.25, 0.50, 0.75, and 1.00 mmol/L). Scale bars, 100 μm. C: Flow cytometry analysis of EpCAM and ITGA6 expression on day 4 under GK15 conditions supplemented with NAC (0, 0.25, 0.50, 0.75, and 1.00 mmol/L). D: Immunofluorescence staining of primordial germ cell markers SOX17 (green), TFAP2C (pink), and BLIMP1 (red) in aggregates on day 4 under GK15 induction conditions supplemented with NAC (0, 0.25, 0.50, 0.75, and 1.00 mmol/L). Nuclei were counterstained with Hoechst (cerulean blue). Scale bars, 50 μm. E: Histogram presenting the average fluorescence intensity of reactive oxygen species expression in aggregates on day 4 under GK15 (grey) and GK15 conditions supplemented with NAC (red). Data are presented as mean ± standard error of the mean. ^***^P < 0.001 by unpaired two-sided Student's *t*-test. The results of six experiments under each condition are shown. F: Heatmap of Pearson correlation coefficients among cells, including EpCAM and ITGA6 double-positive cells in GK15 induction conditions supplemented with NAC (0 mmol/L and 0.5 mmol/L) on day 4, alongside cell types from GSE60138 and GSE67259. Types from GSE67259 are hiPSC, iMeLC, and the BLIMP1-2A-tdTomato and the TFAP2C-2A-EGFP cells (BTAG^+^) induced through iMeLCs (days 2, 4, 6, and 8). Types from GSE60138 are ESC, preinduced cells (pre-induced), day 4 hPGCLCs (PGCLC), and gonadal hPGC (hPGC). Abbreviations: hPGCLCs, human primordial germ cell-like cells; hPSCs, human pluripotent stem cells; NAC, N-acetyl-L-cysteine; hiPSC, human induced pluripotent stem cells; iMeLC, incipient mesoderm-like cells.

The experiments included a control group (0 mmol/L) and treatment groups with 0.25, 0.50, 0.75, and 1.00 mmol/L NAC. The NAC addition started on day 0 of the PGCLC induction process, and the samples were collected for analysis on day 4 of culture (***Fig. 4A***). NAC supplementation did not significantly affect the aggregate volume (***Fig. 4B***). However, the FCM analysis results revealed a marked change in the proportion of the EpCAM and ITGA6 cells, with a particularly pronounced effect at a concentration of 0.5 mmol/L NAC, where the proportion doubled compared with other groups (***Fig. 4C***). Next, we performed immunofluorescence staining on day 4 aggregates to assess the expression of SOX17, TFAP2C, and BLIMP1. The results revealed that the proportion of triple-positive cells increased with increasing concentrations of NAC (***Fig. 4D***). Additionally, ROS levels were compared across aggregates derived from different induction conditions, revealing significantly lower ROS levels after the addition of NAC under GK15 induction conditions than GK15 induction alone (***Fig. 4E***).

In the 0.5 mmol/L NAC group, EpCAM^+^ and ITGA6^+^ cells were sorted by FACS. Then, bulk RNA-seq was performed, and these data were compared with the PGCLC data obtained under GK15 induction conditions and with published data. The Pearson correlation analysis of DEGs across all cell types revealed that the EpCAM and ITGA6 double-positive cells induced under GK15 conditions supplemented with 0.5 mmol/L NAC presented the highest correlation with GK15-induced hPGCLCs, which were also highly correlated with previously published hPGCLC data (***Fig. 4F***). To further investigate the transcriptomic differences between hPGCLCs induced with or without NAC in GK15 medium, we detected almost no DEGs. The *P*-value distribution was nearly uniform, with only a few low *P*-values (***Supplementary Fig. 2A***, available online), suggesting no statistically significant differences in gene expression between the two conditions (GK15 induction conditions with or without 0.5 mmol/L NAC). This finding was further supported by the expression data, where the distributions of gene expression levels were similar between the two conditions (***Supplementary Fig. 2B***, available online), with no apparent shift or clustering indicative of differential expression. We also examined the transcriptional similarity between the GK15-induced hPGCLCs supplemented with 0.5 mmol/L NAC and the N2B27-induced PGCLCs. To compare the overall differences between the two groups, we performed batch correction on the data from the two batches and then conducted a similarity analysis. The results showed that the GK15-induced hPGCLCs supplemented with NAC groups clustered together with the GK15-induced hPGCLC groups (from both batches), while the N2B27-induced hPGCLC groups formed a distinct cluster (***Supplementary Fig. 2C***, available online).

These results revealed that an optimal concentration of NAC effectively increased the *in vitro* induction efficiency of hPGCLCs, indicating that antioxidants play an important role in hPGCLC induction.

## Discussion

We developed a new hPGCLC induction system in which the N2B27 medium, enriched with antioxidant components, was used to induce hPGCLCs. The current study revealed that the N2B27 medium enhanced cell survival in an *in vitro* environment by optimizing oxidative stress responses, improving mitochondrial function, and reducing ROS accumulation, thereby increasing the induction efficiency of PGCLCs. Furthermore, the addition of the antioxidant NAC further validated the crucial role of antioxidant components in PGCLC induction, providing a new strategy and approach for the *in vitro* induction of hPGCLCs.

The induction of germ cell meiosis *in vitro* is a longstanding goal for addressing azoospermia, and this area of research has received significant attention over the years^[40–41]^. Regardless of the specific methodology used, the generation of hPGCLCs represents an essential initial step. However, achieving efficient and large-scale production of hPGCLCs *in vitro* remains a significant challenge. The development of PGCs *in vivo* occurs under unique conditions within the early embryonic environment. This process is governed by intrinsic regulatory mechanisms that determine cell fate, as well as interactions between the embryonic microenvironment and neighboring embryonic cells^[42–44]^. Thus, compared with conventional two-dimensional adherent culture systems, three-dimensional aggregate systems, such as those resembling embryoid bodies, provide a more favorable environment for inducing PGCs^[45]^. These systems recapitulate the microenvironment of early embryonic development more accurately, thereby enhancing the subsequent generation of functional spermatogonia-like cells, spermatocyte-like cells, and even sperm-like cells.

In stem cell experiments, the *in vitro* microenvironment consistently presents challenges for stem cell research and applications. Key factors such as temperature, humidity, oxygen concentration, and pressure are critical, with oxygen concentration being particularly important^[46–47]^. Studies have shown that PSCs exhibit enhanced stability under hypoxic conditions^[15–17]^. However, the optimal oxygen concentration may vary depending on the specific experimental application of PSCs. For example, during the generation of PGCs, the process is not solely dependent on glycolysis; oxidative phosphorylation also plays a crucial role^[33–35]^. Therefore, determining this optimal concentration requires comprehensive testing to minimize oxidative stress and ROS accumulation, which can disrupt energy production and metabolism in cells, ultimately compromising experimental outcomes. In cases where oxidative stress is detected, the controlled addition of antioxidants can mitigate adverse effects, helping cells adapt to oxidative stress and attain homeostasis. This approach ensures the maintenance of cell function and improves the reliability of experimental results.

NAC is a common antioxidant that generally exerts its antioxidant effects by increasing intracellular levels of glutathione. As a precursor to glutathione, NAC is converted into cysteine in the body, which in turn promotes glutathione synthesis. In this way, NAC helps reduce oxidative stress and has demonstrated broad therapeutic potential in the treatment of oxidative stress-related diseases, liver toxicity, poisoning, respiratory diseases, as well as certain neurological and psychiatric disorders^[48]^. The addition of NAC during the hPGCLC induction process may effectively enhance the induction efficiency of hPGCLCs and reduce ROS levels in the culture environment, which is consistent with the results obtained in the N2B27 medium enriched with antioxidants. Therefore, antioxidants play a crucial role in the induction of hPGCLCs. Looking ahead, NAC's antioxidant properties position it as a promising candidate for future clinical applications in reproductive medicine. Given that oxidative stress is a key factor contributing to age-related infertility and idiopathic reproductive disorders, NAC supplementation may help protect germ cells from oxidative damage, preserve oocyte and sperm quality, and improve outcomes in assisted reproductive technologies. Moreover, its ability to modulate the cellular redox state could be beneficial in delaying the age-related decline in fertility and supporting gonadal function in individuals undergoing chemotherapy or other gonadotoxic treatments. Future research is warranted to explore the long-term effects of NAC on human germ cell maintenance, gametogenesis, and overall reproductive health.

Although the current study describes ways to improve the induction rate of PGCLCs under GK15 induction conditions using the antioxidant NAC, the efficiency of hPGCLC induction *in vitro* can be further increased. Future studies may focus on the metabolic pathways involved in PGC development or the cell-cell interactions during early embryonic development. By recapitulating the *in vivo* environment more accurately, the production efficiency and functionality of hPGCLCs may be further improved.

## SUPPLEMENTARY DATA

Supplementary data to this article can be found online.

## References

[1] (2022). Germline stem cells in human. Signal Transduct Target Ther.

[2] (2012). Primordial germ cells in mice. Cold Spring Harb Perspect Biol.

[3] (2009). A signaling principle for the specification of the germ cell lineage in mice. Cell.

[b4] 4Chen D, Sun N, Hou L, et al. Human primordial germ cells are specified from lineage-primed progenitors[J]. Cell Rep, 2019, 29(13): 4568–4582.e5.

[5] (2016). Primordial germ cells: Current knowledge and perspectives. Stem Cells Int.

[6] (2014). Human iPS cell-derived germ cells: Current status and clinical potential. J Clin Med.

[7] (2016). *In vitro* derivation and propagation of spermatogonial stem cell activity from mouse pluripotent stem cells. Cell Rep.

[8] (2016). Complete meiosis from embryonic stem cell-derived germ cells *in vitro*. Cell Stem Cell.

[9] (2022). Germline specification from pluripotent stem cells. Stem Cell Res Ther.

[10] (2015). Generation of fertile offspring from *Kit*^*W*^/*Kit*^*WV*^ mice through differentiation of gene corrected nuclear transfer embryonic stem cells. Cell Res.

[11] (2015). SOX17 is a critical specifier of human primordial germ cell fate. Cell.

[12] (2015). Robust *in vitro* induction of human germ cell fate from pluripotent stem cells. Cell Stem Cell.

[13] (2015). Human primordial germ cell commitment *in vitro* associates with a unique PRDM14 expression profile. EMBO J.

[14] (2010). Oxygen in stem cell biology: A critical component of the stem cell niche. Cell Stem Cell.

[15] (2007). Hypoxia-inducible factors, stem cells, and cancer. Cell.

[16] (2005). Low O_2_ tensions and the prevention of differentiation of hES cells. Proc Natl Acad Sci U S A.

[17] (2009). Continuous hypoxic culturing maintains activation of Notch and allows long-term propagation of human embryonic stem cells without spontaneous differentiation. Cell Prolif.

[18] (2010). The effect of human embryonic stem cells (hESCs) long-term normoxic and hypoxic cultures on the maintenance of pluripotency. In Vitro Cell Dev Biol Anim.

[19] (2014). Roles of reactive oxygen species in the fate of stem cells. Antioxid Redox Signal.

[20] (2015). HISAT: A fast spliced aligner with low memory requirements. Nat Methods.

[21] (2014). FeatureCounts: An efficient general purpose program for assigning sequence reads to genomic features. Bioinformatics.

[22] (2014). Moderated estimation of fold change and dispersion for RNA-seq data with DESeq2. Genome Biol.

[23] (2016). Complex heatmaps reveal patterns and correlations in multidimensional genomic data. Bioinformatics.

[24] (2022). DAVID: A web server for functional enrichment analysis and functional annotation of gene lists (2021 update). Nucleic Acids Res.

[b25] 25Chen D, Liu W, Zimmerman J, et al. The TFAP2C-regulated OCT4 naive enhancer is involved in human germline formation[J]. Cell Rep, 2018, 25(13): 3591–3602.e5.

[26] (2022). BMP4 drives primed to naïve transition through PGC-like state. Nat Commun.

[b27] 27Li L, Dong J, Yan L, et al. Single-cell RNA-seq analysis maps development of human germline cells and gonadal niche interactions[J]. Cell Stem Cell, 2017, 20(6): 858–873. e4.

[28] (2023). Monolayer platform to generate and purify primordial germ-like cells *in vitro* provides insights into human germline specification. Nat Commun.

[29] (2017). Properties of embryoid bodies. Wiley Interdiscip Rev Dev Biol.

[30] (2010). Hypoxia-inducible factors and the response to hypoxic stress. Mol Cell.

[31] (2013). Cellular and molecular mechanisms in the hypoxic tissue: Role of HIF-1 and ROS. Cell Biochem Funct.

[32] (2010). Hypoxia and mitochondrial oxidative metabolism. Biochim Biophys Acta Bioenerg.

[33] (2020). Inherent mitochondrial activity influences specification of the germ line in pluripotent stem cells. Heliyon.

[34] (2019). Metabolic regulation of pluripotency and germ cell fate through α-ketoglutarate. EMBO J.

[35] (2019). Alpha-ketoglutarate: A "magic" metabolite in early germ cell development. EMBO J.

[36] (2006). Mitochondrial ROS-induced ROS release: An update and review. Biochim Biophys Acta Bioenerg.

[37] (2024). Reactive oxygen species and cell signaling. Review. Biochim Biophys Acta Mol Cell Res.

[38] (2022). GlyNAC (glycine and N-acetylcysteine) supplementation in mice increases length of life by correcting glutathione deficiency, oxidative stress, mitochondrial dysfunction, abnormalities in mitophagy and nutrient sensing, and genomic damage. Nutrients.

[39] (2016). A minireview on *N*-acetylcysteine: An old drug with new approaches. Life Sci.

[b40] 40Ishikura Y, Ohta H, Sato T, et al. In vitro reconstitution of the whole male germ-cell development from mouse pluripotent stem cells[J]. Cell Stem Cell, 2021, 28(12): 2167–2179. e9.

[41] (2020). Long-term expansion with germline potential of human primordial germ cell-like cells *in vitro*. EMBO J.

[42] (2010). Germ cell specification in mice: Signaling, transcription regulation, and epigenetic consequences. Reproduction.

[43] (2016). The germ cell fate of cynomolgus monkeys is specified in the nascent amnion. Dev Cell.

[44] (2009). Germ cell specification in mice. Curr Opin Genet Dev.

[45] (1995). *In vitro* differentiation of embryonic stem cells. Curr Opin Cell Biol.

[46] (2015). Concise review: The role of oxygen in hematopoietic stem cell physiology. J Cell Physiol.

[47] (2021). Discrepancies on the role of oxygen gradient and culture condition on mesenchymal stem cell fate. Adv Healthc Mater.

[48] (2008). *N*-acetylcysteine for antioxidant therapy: Pharmacology and clinical utility. Expert Opin Biol Ther.

