# Supplementary material for: Improving in vitro induction efficiency of human primordial germ cell-like cells using N2B27 or NAC-based medium
Source: J Biomed Res. 2025 Apr 10;39(6):587–600. doi: 10.7555/JBR.38.20240433 (PMC12683510; doi:10.7555/JBR.38.20240433)
Supplement: Supplementary file 1 — Supplementary data to this article can be found online. [file jbr-39-6-587-Supplementary.pdf]

Available online at www.jbr-pub.org.cn

Open Access at PubMed Central

Journal of Biomedical Research, 2025 39(6): 1–3

JBR

Supplementary Data

Improving *in vitro* induction efficiency of human primordial germ cell-like cells using N2B27 or NAC-based medium

Gege Yuan[1,△], Jiachen Wang[1,△], Shuangshuang Qiu[1], Yunfei Zhu[1], Qing Cheng[2], Laihua Li[1], Jiahao Sha[1], Xiaoyu Yang[3,✉], Yan Yuan[1,✉]

[1]*State Key Laboratory of Reproductive Medicine and Offspring Health, Nanjing Medical University, Nanjing, Jiangsu 211166, China;*
[2]*Women's Hospital of Nanjing Medical University, Women and Children's Healthcare Hospital, Nanjing, Jiangsu 211100, China;*
[3]*State Key Laboratory of Reproductive Medicine, Clinical Center of Reproductive Medicine, the First Affiliated Hospital of Nanjing Medical University, Nanjing, Jiangsu 210029, China.*

Differentially expressed genes in EpCAM/ITGA6 double-positive cells and d4BTAG$^+$ cells are listed in ***Supplementary Table 1*** (available online). GO enrichment results for EpCAM-positive/ITGA6-weakly positive (+/–), EpCAM/ITGA6 double-negative (–/–), and EpCAM/ITGA6 double-positive (+/+) cells are provided in ***Supplementary Table 2*** (available online). GO and KEGG enrichment analyses of differentially expressed genes in PGCLCs induced under GK15 conditions versus N2B27 conditions are shown in ***Supplementary Table 3*** (available online).

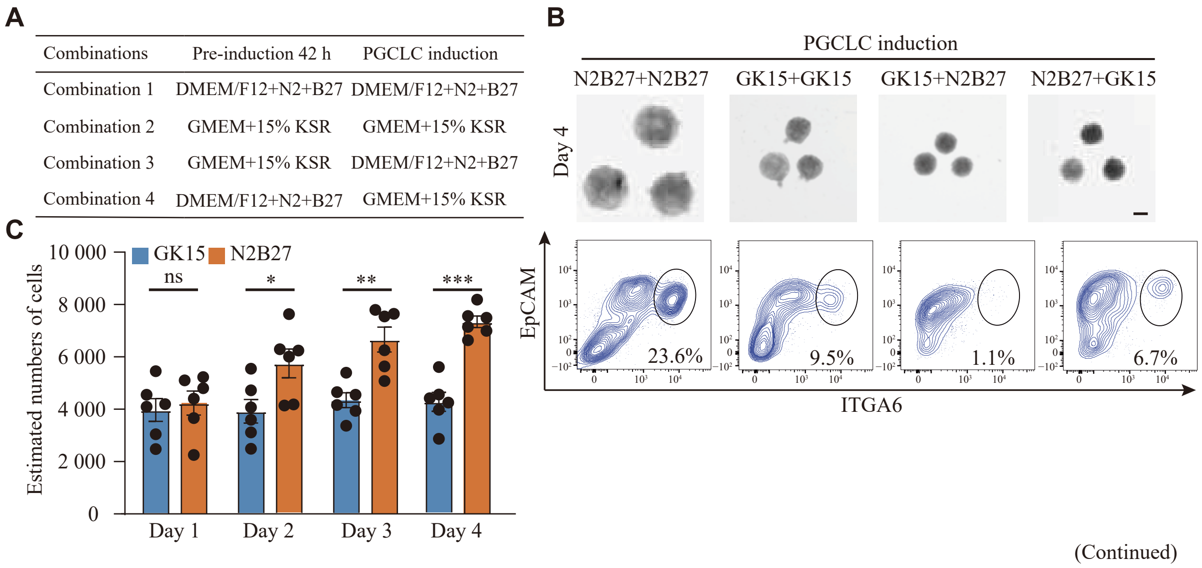

(Continued)

△These authors contributed equally to this work.

✉Corresponding authors: Xiaoyu Yang, State Key Laboratory of Reproductive Medicine, Clinical Center of Reproductive Medicine, the First Affiliated Hospital of Nanjing Medical University, 300 Guangzhou Road, Nanjing, Jiangsu 210029, China. E-mail: yxy1921@163.com; Yan Yuan, State Key Laboratory of Reproductive Medicine and Offspring Health, Nanjing Medical University, 101 Longmian Avenue, Nanjing, Jiangsu 211166, China. E-mail: yuanyan@njmu.edu.cn.

Received: 08 December 2024; Revised: 23 March 2025; Accepted: 26 March 2025; Published online: 10 April 2025

CLC number: R321.1, Document code: A

The authors reported no conflict of interests.

This is an open access article under the Creative Commons Attribution (CC BY 4.0) license, which permits others to distribute, remix, adapt and build upon this work, for commercial use, provided the original work is properly cited.

© 2025 by Journal of Biomedical Research.

https://doi.org/10.7555/JBR.38.20240433

(Continued)

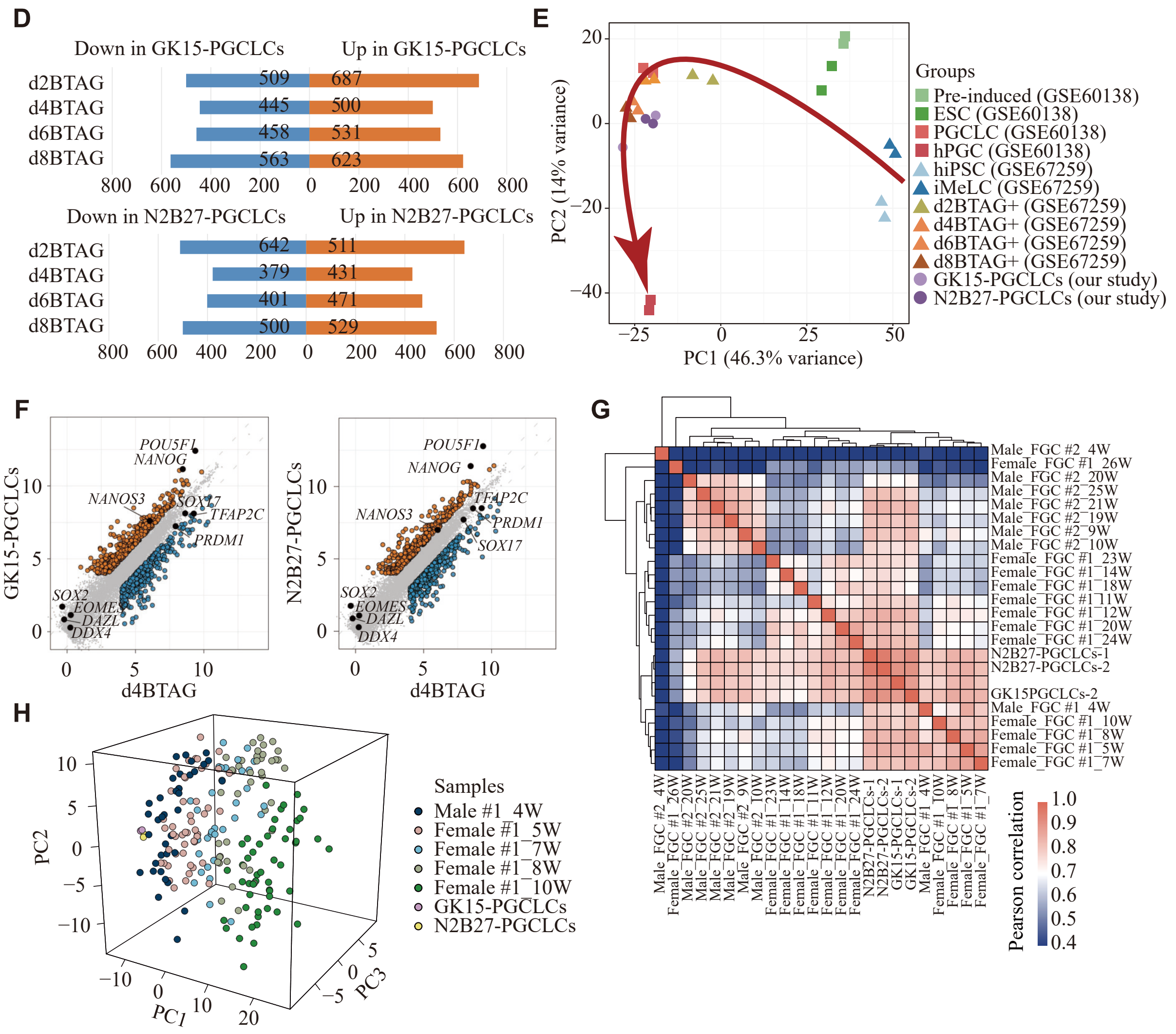

Supplementary Fig. 1 **Induction of aggregates with PGCLC characteristics *via* the N2B27 medium.** A: The table presents the combinations of different basal media. B: Representative bright-field images of floating aggregates on day 4 during hPGCLC induction under four combination conditions. Scale bars, 100 µm (upper). Flow cytometry analysis of EpCAM and ITGA6 expression on day 4 under four combinations (lower). C: Histogram presenting the estimated cell numbers during the hPGCLC induction from day 1 to day 4 under floating aggregates in GK15 (blue) or N2B27 (orange) conditions. *$P < 0.05$, **$P < 0.01$, and ***$P < 0.001$ (unpaired two-sided Student's *t*-test). The error bar indicates the standard error of the mean. Results from six experiments under each condition are shown. D: Numbers of differentially expressed genes between EpCAM and ITGA6 double-positive cells under GK15 (up) and N2B27 (bottom) induction conditions on day 4 and cells at different induction times from GSE67259. E: Principal component analysis of the indicated cell types described in *Fig. 1F*. The cells (color-coded as indicated) are plotted in a two-dimensional space defined by principal components (PC) 1 and 2. F: Scatter-plot comparison of the averaged gene-expression levels between EpCAM and ITGA6 double-positive cells under GK15 (left) and N2B27 (right) induction conditions on day 4 and the BLIMP1-2A-tdTomato and the TFAP2C-2A-EGFP cells on day 4 (d4BTAG) from GSE67259. Orange and blue circles indicate up- and down-regulated genes. G: Heatmap of correlation coefficients among cells, including EpCAM and ITGA6 double-positive cells under GK15 and N2B27 induction conditions on day 4, alongside cell types from GSE86146. H: Principal component analysis of the indicated cell types, including EpCAM and ITGA6 double-positive cells under GK15 and N2B27 induction conditions on day 4, alongside cell types from GSE86146. The cells (color-coded as indicated) are plotted in a three-dimensional space defined by PC1, PC2, and PC3. Abbreviations: hPGCLCs, human primordial germ cell-like cells; EpCAM, epithelial cell adhesion molecule; ITGA6, integrin alpha-6; BLIMP1, B lymphocyte-induced maturation protein 1; TFAP2C, transcription factor AP-2 gamma.

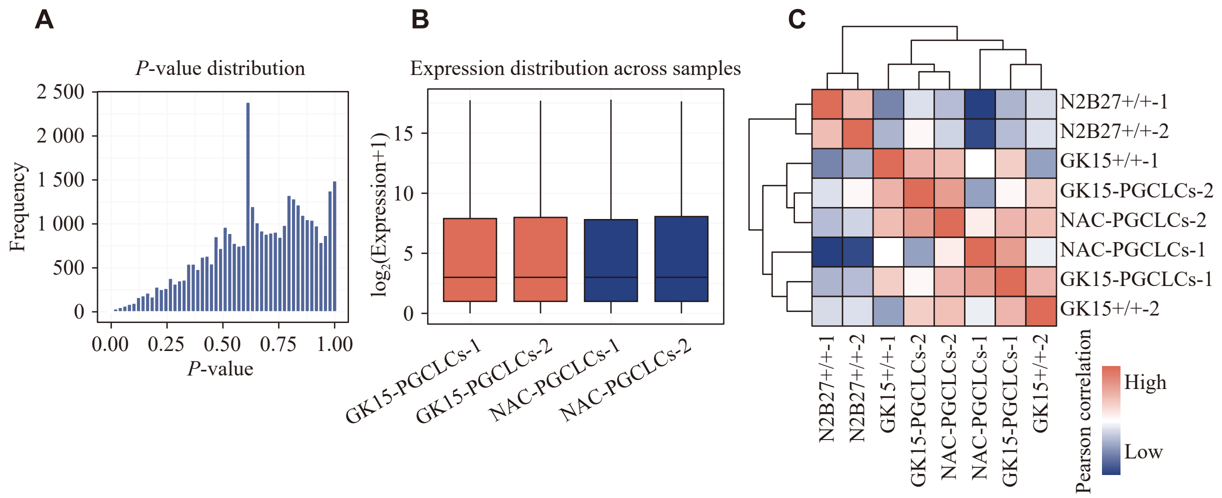

Supplementary Fig. 2 **The transcriptional characteristics of NAC-induced hPGCLCs.** A: *P*-value distribution obtained from DESeq2 analysis. B: Gene expression distribution of hPGCLCs under GK15 induction conditions (red) and GK15 induction conditions supplemented with 0.5 mmol/L NAC (blue). C: Heatmap of correlation coefficients among cells, including EpCAM and ITGA6 double-positive cells in GK15 induction conditions (named GK15+/+), N2B27 induction conditions (named N2B27+/+), and GK15 induction conditions supplemented with 0 or 0.5 mmol/L NAC (named GK15-PGCLCs, NAC-PGCLCs) on day 4. Abbreviations: hPGCLCs, human primordial germ cell-like cells; NAC, N-acetyl-L-cysteine; EpCAM, epithelial cell adhesion molecule; ITGA6, integrin alpha-6.
